# Floods, Hurricanes, and Other Catastrophes: A Challenge for the Immune System of Livestock and Other Animals

**DOI:** 10.3389/fvets.2020.00016

**Published:** 2020-01-31

**Authors:** Joel F. Filipe, Valentina Herrera, Giulio Curone, Daniele Vigo, Federica Riva

**Affiliations:** Dipartimento di Medicina Veterinaria, Università degli Studi di Milano, Milan, Italy

**Keywords:** climate change, livestock, immune system, global warming, inflammatory response

## Abstract

Climate change involves different dramatic phenomena including desertification and wildfires, severe storms such as hurricanes and blizzards, increased sea levels resulting in flooding coastal cities and rise of atmospheric CO_2_ concentration. The alteration of the climate in a specific region affects the life of indigenous animals and humans. The climate changes influence living beings both directly and indirectly. The immune system of animals dramatically suffers the climate instability, making animals more susceptible to infectious and not infectious diseases. Different species of livestock animals respond with similar mechanisms to global warming, but some of them are more susceptible depending on their age, metabolism, and genetic conditions. The selection and study of autochthonous species and breeds, more easily adapted to specific environmental conditions could be an interesting strategy to face livestock rearing in the future.

## Introduction

Climate changes dramatically increased over the past 20 years. First of all, a global warming has caused the increase of 1°C of the Earth's global surface temperature compared to the average in the mid XX century (1). This phenomenon leads to weather anomalies that drastically affect indigenous plant and animal life. The unstable weather patterns lead to desertification and wildfires, severe storms such as hurricanes and blizzards, increased sea levels resulting in flooding coastal cities and rise of atmospheric CO_2_ concentration (2). The result of all these events is that warm areas of the planet can cross cool seasons and cold areas of the planet can experience very hot temperatures. Plants and animals are able to face unexpected acute modifications of their environment thanks to their resilient ability, but when such a modification becomes chronic, or too extreme, they can be seriously affected. Moreover, the climate change can spread pathogens and pathogen vectors in new areas where plant and animals are not equipped to fight them. Finally, the climate changes and all the following weather anomalies represent a strong stress for the immune system and finally can put at risk the health of humans and animals. The immune system of animals can be affected both directly and indirectly by climate changes. For example, high environmental temperature directly affects the immune system causing an increase of the body temperature and indirectly, causing a reduction of the food intake, limits the energy sources for the immune responses that are known to be very energy consuming. This review aims to update all the knowledge's on immune system alterations caused by different phenomena of the climate change, focusing on the need to consider multi-stressor effects when evaluating climate change impacts upon animal immune system.

## Global Warming and Immune System of Mammals and Avian Livestock

Global warming is the main and strongest phenomenon associated to climate change. The Earth's temperature is increasing by 0.2°C per decade and it is estimated that by 2100 it will be increased by 1.4–5.8°C (3). Most of the studies on climate change effects on immune system focus on the increasing temperatures worldwide. In fact, livestock animals are homoeothermic animals, they can maintain a constant body temperature through the exchange of heat produced by their metabolism and the external environment (4). Farm animals have a thermoneutral zone (TNZ) between 16 and 25°C where they manage to maintain a body temperature between 38.4 and 39.1°C. When the external temperature reach values below or above the TNZ, animals have to actively adapt by playing out physiological, morphological and behavioral adaptation mechanisms (5, 6). The majority of the response to heat stress are common to different species of vertebrates.

The first consequence that heat stress has on livestock is the decrease in daily food intake. Indeed, high environmental temperatures negatively affect the hypothalamic appetite center by reducing food intake (7). The reduction of food intake is a mechanism that aims to reduce the production of heat due to the metabolism, both in the polygastric and in the monogastric animals (8, 9).

Among farm animals, goats are the ruminants that better resist to temperature variations (10). Despite the decrease in dry matter intake (DMI) in goats, there is only a slight reduction in milk production (3–10%), compared to other dairy ruminants. The reduction of milk fat is not accompanied by a reduction in glucose levels or an increase of insulin levels as in cows (11, 12). In lactating cows the food intake begins to decrease at an environmental temperature around 25–26°C and the reduction can reach the 40% when it reaches 40°C (13). At these temperatures, the food intake in dairy goats can be reduced by 22–35% (11) and in buffaloes by 8–10% (14). Also in pigs the caloric intake is reduced when they are subjected to thermal stress (9). Heat stress also affects poultry's behavior. At high temperature the time devoted by chickens to nourishment decreases, they drink less, spend more time on the care of their wings, moving, and walking less (15). The nutrients administered with feed to poultry provide the substrates for cell proliferation and differentiation (also leukocytes); moreover, they can act as immunomodulators. Antigens supplied with the diet stimulate the production of immunoglobulin's in the bursa (16–18). Food intake is than necessary for a correct and efficacious immune response (19).

Another common response to heat stress in different farm animal species (cattle, goats, sheep, pig, poultry) is the increase in peripheral cortisol levels (19–22). Indeed, the heat stress causes the activation of the two axes hypothalamic-pituitary-adrenal (HPA) and sympathetic-adrenal-medullar (SAM), with the final release of cortisol and catecholamines that are known to influence the biology of leukocytes. Lymphocytes, monocytes, macrophages, and granulocytes have receptors for cortisol and catecholamines that mediate a negative alteration of their trafficking, proliferation, cytokine secretion, immunoglobulin (Ig) production, and cytotoxic activity (23).

Poultry subjected to heat stress showed a reduction of the volume and weight of lymphoid organs, low levels of circulating, and intestinal Ig and reduced phagocytosis (24–26). The reduced capability of leukocytes to produce proteins (cytokines and Ig) was mainly due to the phosphorylation of EF2 protein that blocks the protein production by altering the elongation phase of translation (21).

In dairy cows the activation of HPA and SAM axes induce the release of glucocorticoids and catecholamines and the following reduction of cytokine levels, particularly IL-4, IL-5, IL-6, IL-12, IFN-γ, and TNF-α (19). High levels of cortisol lead to immune suppression, overall on T cell compartment, cytokine production, and phagocytosis. Also, catecholamines negatively affect the immune system by inhibiting IL-12 production and promotion of IL-10 release, finally promoting a Th2 response. The hyperthermia concurs to the inhibition of Th1 response, favoring the Th2 mediated humoral response (19). Cortisol aggravates the immunosuppression by inhibiting the activation of T cell, the production of cytokines and the phagocytosis (19). In these conditions of immunosuppression vaccination of animals is inefficacious and some latent viruses can reactivate (19). Once cortisol binds to glucocorticoid receptors on the surface of leukocytes, the activation of proinflammatory transcription factors AP-1 and NFkB on the MAPK signaling pathway (19). The immunosuppression involves also the downregulation of complement system component (19). In dairy cows the majority of the data describes after prolonged heat periods an increase of total leukocytes with an altered differential count, where neutrophils increase, recruited from the bone marrow by cortisol, and lymphocytes decrease (19). Cortisol induces a downregulation of L-selectin which, in turn, reduces the activity of neutrophils and upregulates heat shock proteins (HSP) (27–29). It is interesting to note that if the heat stress occurs during the dry off period, the following lactation is compromised and the animal is more susceptible to production diseases, suggesting the existence of a sort of immunologic memory in the innate immune cells, likely mediated by epigenetic mechanisms (29, 30). Moreover, the reduced production of Ig following the heat stress, compromise the passive immunity transfer from the cow to its calf (19).

Another response common in vertebrates after heat stress is the production of HSPs. In poultry the attempt to maintain a correct body temperature induces the production of reactive oxygen species (ROS) and a consequent oxidative stress in the animal. The oxidative stress stimulates the liver of chicken to produce increased levels of HSPs to protect the tissues against the free radicals (31, 32). In dairy cattle increased levels of HSPs have been described (19). HSP70 and HSP90 activate an inflammatory response mediated by TLR4 (19). Dairy ruminants can activate antioxidant pathways to counteract the negative effects due to excessive production of reactive oxygen species (ROS). Indeed, high levels of catalase, superoxide dismutase (SOD), glutathione reductase (GSH) and malondialdehyde (MDA) have been observed in buffaloes and cows in summer months (33, 34). In sheep subjected to heat shock HSPA2, a member of HSP70 family, resulted upregulated. HSPA2 is known to induce the production of IL-1β that through IL-1R1, also upregulated in sheep under heat stress, can trigger an inflammatory response (35). Also, pigs subjected to prolonged heat stress develop signs of inflammation at the hepatic level (36). Indeed, their hepatocytes present an upregulation of HSPs and TLRs genes, signs of oxidative stress (increased GSH:GSSG ratio) and increased apoptosis (upregulation of PDIA3, P4HB, IRF9, VIM, NDGR2) (37, 38). These responses are partly directly induced by the high environmental temperature and partly indirectly by the reduced feed intake (36).

Data on the effect of global warming on the immune system of livestock sometimes are in contrast and this could be due to genetic differences of different breeds under study. For example, in poultry it has been described that different breeds of broilers and hens respond differently to heat stress (15, 39). In general, autochthonous breeds can adapt easily to environmental stress compared to others. Salem Black goats are known to better adapt to high temperature compared to Malabari and Osamabadi goats, showing lower levels of HSP70 after a prolonged period of high environmental temperature compared to the other two breeds (40). On the other hand, autochthonous animals reared in different condition compared to their usual ones, can develop dramatic stress responses. For example, the Tharpakar cow, a dual-purpose breed native to arid zone, shows an immunosuppression with and increase incidence of mastitis cases when reared in hot and humid environment (41). Different studies aimed to improve the resilience of livestock, identified genes associated to heat resistance such as heat shock 27 kD associated protein 1 (HSPBAP1) in goats, an inhibitor of HSP27. SNPs within HSPBAP1 gene were associated to susceptibility/resistance to heat stress (42). Other studies in cattle and chicken identified genes involved in different mechanisms such as immune response (interleukins and cluster differentiation markers), metabolism (NADH), remodeling of mammary gland and central nervous system functions confirmed the complexity of heat shock response in animals (43, 44). All these studies evidence that the resistance to high temperature negatively correlate to the production level of the animals (45).

## Global Warming and Immune System of Fish Livestock

Human activities can have a huge impact on climate changes, influencing directly, and/or indirectly not only terrestrial environments but also aquatic ones. In aquatic environments those changes can be chemical (e.g., acidity, salinity, oxygen levels), physical (e.g., temperature), or biological (e.g., algae growth). These modifications of the environment affect the animals, in particular their immune system, impairing their capability of protection against pathogens (46–49).

Motile organisms, like fish, can be tolerant to some environmental changes or can escape from those alterations moving from one place to another, but aquacultured fish raised in sea cages, are not able to relocate in order to avoid ecological alterations. Those changes can be considered stressors, and in natural environments, those do not normally occur alone but in combination (49–51).

Water temperature can deeply affect fish immune system. Acute and chronic changes in temperature have also different impacts on animals, being short term episodes compensated by processes such as heat shock protein response, while chronic temperature variations are less likely to be solved by such responses (52–55).

Some studies suggest that it polarize the immune responses: at low temperatures fish may rely more on innate immune system, while at higher temperatures is the adaptive immunity that is more efficient (56–60). Pattern recognition by glucan binding proteins was predominant in perch (*Perca fluviatilis*) acclimated at lower temperatures, while, opsonin was more effective at higher temperatures (61).

A common phenomenon associated to the response of immune system to high environmental temperature (water temperature) is the increase of the antibody levels. This has been reported in several studies with different fish species (60, 62–73), where high temperatures seem to boost antibody levels indicating that a potential increment of oceans temperatures could actually help protecting some aquatic species against pathogens (73, 74).

Also, innate immunity of fishes is affected by water temperature; in South Asian carp (*Catla catla*) Toll Like Receptors (TLRs) and NOD-like receptors are modulated both by cold or warm water. TLR2, TLR4 and NOD2 expression increases with higher water temperatures, while TLR5 and NOD1 expression increases at both high and low temperatures (69).

In three-spined sticklebacks (*Gasterosteus aculeatus*) granulocyte respiratory burst activity and lymphocyte proliferation were inhibited when maintained for short period at high water temperatures and enhanced at low water temperatures. When the animals were exposed to a ‘heat wave' of 28°C for 2 weeks, long lasting immune disorders occurred resulting in the impairment of the immune system and the following spread of infectious diseases among those fish populations (70).

Temperature in gene expression changes of immune molecules. In farm raised Atlantic cod (*Gadus morhua*), cold water (up to 16°C) caused an increase of b2-M, MHCI, and IgM mRNA expression; on the contrary warmer raising water induced a downregulation of the same genes, being only IL-1β upregulated at high water temperatures (48). By the other hand, in the skin of Atlantic salmon (*Salmo salar*) low temperatures induce IL-1β, IL-8, and TNF-α upregulation (73).

## Alterations of Salinity and Acidity of Oceans and Immune System of Fish Livestock

The climate change has induced modification of water environment in terms of salinity and pH.

Ocean acidity can change as a result of natural environmental sources (71), but different studies consider anthropogenic activities as the main contributors for water acidification. Approximately one third of the CO_2_ released since the industrial revolution has been taken up by the oceans (72), leading to the drop of pH (72). Recent studies estimated an average decrease in ocean pH of 0.1 in the last two centuries (75). Atlantic halibut (*Hippoglossus hippoglossus*) exposed to different water temperatures and pH levels showed signs of inflammatory responses with increased expression of complement component C3 and fibrinogen β chain precursor at high CO_2_ concentrations (reduced pH), without the influence of the temperature, suggesting that these changes are directly a consequence of the decrease of pH (74, 76).

In some species (*Squalius carolitertii* and *Squalius torgalensis*) the combination of low pH and high temperature of the water triggers the downregulation of Interferon-induced guanylate-binding protein 1 (GBP1) gene in liver, an antiviral and antimicrobic factor (49).

The alteration of the salt composition of waters is mainly due to the increased evaporation and strongly influences the life of fishes (77). In turbot (*Scophthalmus maximus*) Hsp70 and IgM expression was correlated to both temperature and salinity, temperature being the dominant factor (62). Freshwater fish are naturally tolerant to salinity alterations, but in seawater the complex variations of salinity can alter fish immune functionality, especially in stenohaline fish (78). A study developed in Nile tilapia (*Oreochromis niloticus*), a freshwater fish that prefers brackish waters, demonstrated that slowly increasing salinity in the environment did not have any significant impact on monocyte and lymphocyte number and phagocytotic process, yet a continuous decrease in water salinity was correlated with an increment in leukocyte number and phagocytosis (79, 80). In a different tilapia species (*Oreochromis mossambicus*), the increased salinity water caused a gain in lysozyme activity in plasma and head kidney homogenate (81).

A study on gilthead sea bream (*Sparus aurata*), a commonly aquacultured species with a wide range of salinity tolerance, demonstrated that hyper-saline water highly increases IgM production, while a decrease on water salinity do not (82). In another work, pipefish (*Syngnathus typhle*) kept in high salinity conditions and infected with *Vibrio* spp. presented significantly higher phagocytosis values compared to controls. When the fishes remained for a longer period in high salinity environment increased energy was required for osmoregulation leading to both lymphocyte and monocyte proliferation reduction, suggesting that during longer high salinity periods animals can be immunocompromised (47).

Water oxygen levels is another important parameter for aquatic animals' survival, and this parameter is clearly related to water temperature, salinity, and ionic concentration. Among the few studies published, hypoxia, and water temperature were investigated on Atlantic cod (*Gadus morhua*), and the expression of HSP70 was significantly higher in hypoxic conditions and at low temperatures (51). Macrophages of Atlantic salmon *in vitro* stimulated with poly I:C (TLR ligand) showed a significant increase of IFN-α mRNA levels in non-hypoxic conditions compared to normoxic conditions. This difference suggests that chronic hypoxia can modulate the innate immune response, altering the susceptibility of those animals to infections (83).

## Alteration of Water Cycle and Immune System of Livestock Animals

The increased salinity of the oceans has been demonstrated to alter the water cycle of the earth leading to dramatic phenomena such as rainfalls, floods, and dust storms (77). The consequence of the alteration of the water cycle is that “arid regions have become drier and high rainfall regions have become wetter” (84).

So, the climate change, including alteration of water cycle and atmospheric CO_2_, concurs to the modification of the plant composition and accordingly to the reduction of food quality and quantity (85). The poor quality and low quantity of food negatively influences the immune response of animals, that is highly energy demanding and continuously requires adequate immune stimulation (19, 85). In these conditions, animals are more susceptible to infections and infestations.

Moreover, the alterations of the climate conditions allow the worldwide distribution of vectors of infectious diseases once endemic in specific regions. Burden of vector borne diseases increased in the last years depending on different factors: short life cycle of the vectors, reduction of incubation period, increased number of vector populations and extension of the times of transmission of the pathogen. All this factors are deeply influenced by the environment in particular temperature and water/humidity. Indeed both mosquitos and ticks, the major vectors, are highly susceptible to global warming, floods, and droughts. In particular the increased temperatures favor the spread of mosquitos in Northern latitudes where they find a suitable niche for reproduction and can overwinter, whereas in the tropical areas very high temperatures and the alternation of heavy rainfalls and droughts exacerbate the incidence of vector borne diseases by shortening the life cycle of vectors and promoting the host-pathogen interaction due to the livestock overcrowding at the water pools in dry seasons (86–88). Based on this, prediction models suggest a wide spread of vector borne diseases such as Rift Valley fever and Malaria (89, 90). Regarding tick vectors, their movement toward Northern region has been registered. For example *Ixodes ricinus* has been documented in Sweden and Russia, whereas *Ixodes persulcatus* in subarctic regions (91–93). The climate change can also negatively affect the spread of vector borne diseases. Indeed, the excessive temperature rise and prolonged dry period in subtropical and tropical areas can reduce the survival and reproduction rate of specific tick species such as *Riphicephalus sanguineus*. For the same reason epidemiological models predict that Leishmaniosis will decrease in the future (90).

The spread of vector borne diseases can induce immunosuppression in livestock and humans, favoring a further circulation of diseases (19). This add up to the immunosuppression caused by heat stress and sudden changes of temperatures, aggravating the susceptibility to infections (19, 94).

Pig farming is largely developing in tropical areas where climate change causes very hot and humid summers. These conditions challenge the immune system as previously described by activating the HPA axis and resulting in the immune suppression of the animals. The poor hygiene conditions typical of the tropical areas favor the spread of pathogens such as *Salmonella* and *Isospora* among pigs also in northern regions (95, 96).

Finally, the increase frequency of dust storm can impact on animal health. Strong winds transport dust with a complex and variable composition around very wide areas of the world. Dust is mainly made up of silicon dioxide, aluminum oxide, iron and titanium oxides, calcium and magnesium oxides, sodium and potassium oxides (97). It can also contain microorganisms such as bacteria, fungi, and viruses (98–100). The small dimension of the particles (PM 0.1 and PM 2.5) is the main responsible of the tissue damages, causing apoptosis, autophagy, and oxidative stress in the airway cells (101, 102).

## Conclusions

**Figure 1:**
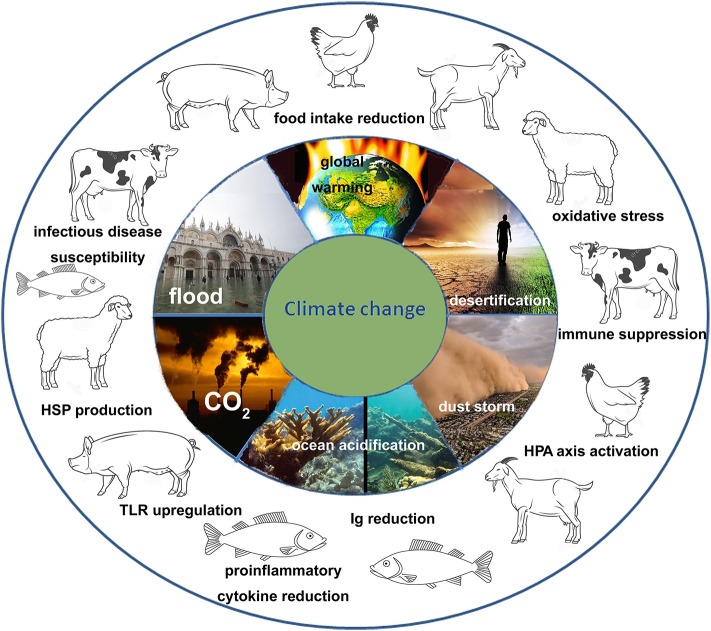
Scheme of the impact of climate change on the immune system of livestock animals. Climate change is a complex phenomenon that include different dramatic event such as global warming, desertification, alteration of water cycle leading to floods and droughts, increase concentration of atmospheric CO_2_, acidification, and alteration of the salinity of the oceans. All these events deeply affect the immune system of livestock species (mammals, avian, and fishes) both directly and indirectly. Many of the responses are common to different animals such as the activation of the HPA axis with the release of cortisol (that is an immune suppressor), production of HSPs in response to the oxidative stress, reduction of Ig production and increased susceptibility to infectious diseases.

Climate change includes several dramatic phenomena such as global warming, rise of atmospheric CO_2_ concentration, alteration of salinity and pH of oceans, reduction of O_2_ concentration in waters that lead to desertification and wildfires, severe storms such as hurricanes and blizzards, increased sea levels resulting in flooding coastal cities. All these phenomena are tightly linked to one another (Figure 1). So the climate change should be analyzed as a very complex problem and should be faced by an integrated strategy at different levels. Climate change impacts on production and reproduction of livestock causing important economic losses, being the high yield animals (with an accelerated metabolism and a genetic selection based on production) the less resilient and the most affected by the environment modifications. But it also impacts on the immune system of the animals inducing immune suppression and increasing their susceptibility to infections. The spread of vector borne diseases promoted by climate change also contribute to the impairment of the animal welfare. On the other hand, the intensive farming of livestock contributes to worsen the global warming, mainly by the emission of green-house gases such as CO_2_, CH_4_, and N_2_O (livestock sector concurs directly and indirectly with 18% of green-house gases emissions) (103). Given that climate change will progressively reduce the quantity and the quality of food for humans and animals, one strategy for future livestock management could be the valorization of autochthonous livestock breeds known to be highly resilient and disease resistant, to have low dietary needs (they have good production levels also with a frugal ration) and to produce high quality products (104).

## Author Contributions

JF, VH, GC, DV, and FR discussed the organization of the manuscript, drafted the manuscript and commented on the manuscript.

### Conflict of Interest

The authors declare that the research was conducted in the absence of any commercial or financial relationships that could be construed as a potential conflict of interest.
